# Comprehensive identification and functional characterization of *GhpPLA* gene family in reproductive organ development

**DOI:** 10.1186/s12870-023-04590-4

**Published:** 2023-11-29

**Authors:** Mingyang Wang, Dingyan Tian, Tengyu Li, Jingwen Pan, Chenlei Wang, Lanxin Wu, Kun Luo, Zhenyu Mei, Jinwei Liu, Wei Chen, Jinbo Yao, Yan Li, Fuxin Wang, Shouhong Zhu, Yongshan Zhang

**Affiliations:** 1 National Engineering Research Center of Cotton Biology Breeding and Industrial Technology, Institute of Cotton Research, Chinese Academy of Agricultural Science, Anyang, Henan 455000 China; 2 Zhengzhou Research Base, State Key Laboratory of Cotton Biology, Zhengzhou University, Zhengzhou, Henan 450001 China; 3 College of Agronomy, Tarim University, Alar, Xinjiang 843300 China; 4 College of Advanced Agricultural Science, Zhejiang A&F University, Hangzhou, Zhejiang 311300 China; 5 College of Life Sciences, Hebei University, Baoding, Hebei 071002 China

**Keywords:** Cotton, *pPLAs*, Evolutionary analysis, Functional characterization, VIGS, Reproductive organ

## Abstract

**Background:**

Phospholipases As (*PLAs*) are acyl hydrolases that catalyze the release of free fatty acids in phospholipids and play multiple functions in plant growth and development. The three families of *PLAs* are: *PLA1*, *PLA2* (*sPLA*), and patatin-related *PLA* (*pPLA*). The diverse functions that *pPLAs* play in the growth and development of a broad range of plants have been demonstrated by prior studies.

**Methods:**

Genome-wide analysis of the *pPLA* gene family and screening of genes for expression verification and gene silencing verification were conducted. Additionally, pollen vitality testing, analysis of the pollen expression pattern, and the detection of POD, SOD, CAT, MDA, and H_2_O_2_ were performed.

**Result:**

In this study, 294 *pPLAs* were identified from 13 plant species, including 46 *GhpPLAs* that were divided into three subfamilies (I-III). Expression patterns showed that the majority of *GhpPLAs* were preferentially expressed in the petal, pistil, anther, and ovule, among other reproductive organs. Particularly, *GhpPLA23* and *GhpPLA44*, were found to be potentially important for the reproductive development of *G. hirsutum*. Functional validation was demonstrated by VIGS which showed that reduced expression levels of *GhpPLA23* and *GhpPLA44* in the silenced plants were associated with a decrease in pollen activity. Moreover, a substantial shift in ROS and ROS scavengers and a considerable increase in POD, CAT, SOD, and other physiological parameters was found out in these silenced plants. Our results provide plausibility to the hypothesis that *GhpPLA23* and *GhpPLA44* had a major developmental impact on cotton reproductive systems. These results also suggest that *pPLAs* are important for *G. hirsutum’s* reproductive development and suggest that they could be employed as potential genes for haploid induction.

**Conclusions:**

The findings of the present research indicate that *pPLA* genes are essential for the development of floral organs and sperm cells in cotton. Consequently, this family might be important for the reproductive development of cotton and possibly for inducing the plant develop haploid progeny.

**Supplementary Information:**

The online version contains supplementary material available at 10.1186/s12870-023-04590-4.

## Introduction

Plant reproductive development has gained a significant attention of researchers and breeders being a crucial aspect of plant life cycle. In the reproduction of flowering plants, double fertilization is an intricate vital process that serves a vital role in the production of offsprings [1]. Exploring the gene families involved in double fertilization is essential for understanding this mechanism, which is not only shared by all angiosperms but also essential for plant genetics and breeding. Pollen-specific phospholipase *MTL* (*PLA*) naturally contains frame-shifting mutations that create haploids by rearranging sperm membranes and interfering with double fertilization, it has been discovered to have a significant impact on crop development and breeding [2, 3]. It is essential to recognize and functionally describe *PLA* gene family in order to understand the *PLA* gene’s role in plant reproductive development.

Phospholipases (*PLAs*) are a family of ubiquitous proteins that cleaves various bonds in phospholipids to maintain membrane lipid homeostasis stability and also play an important role in signal transduction [4, 5]. *PLAs* are extensively dispersed and are categorized into three classes (A, C, and D) according to the particular cleavage sites that the hydrolases target [6]. Plant *PLAs* are classified into three families: *PLA1*, *PLA2* (*sPLA*), and patatin-related *PLA* (*pPLA*). These *PLAs* hydrolyze either sn-1 or sn-2 acyl groups. Interestingly, *pPLAs* showed activity at both sn-1 and sn-2 sites, indicating the unique characteristics of *pPLAs*, while *PLA1* and *sPLA* only target sn-1 and sn-2, respectively. [7] In *Arabidopsis thaliana*, *pPLAs* are divided into three subfamilies (*pPLA-I*、*pPLA-II* and *pPLA-III*) [7, 8]. In *Arabidopsis*, there is strong expression of *pPLA-IIε* and *pPLA-IIδ* in the roots and root hairs, as well as *pPLA-IIδ* in the leaves and cotyledons. The remaining *pPLAs* are primarily expressed in pollen (*pPLA-I*), seeds (*pPLA-I*, *pPLA-IIβ*, and *pPLA-IIIα*), and roots (*pPLA-IIIγ* and *pPLA-IIIδ*). Only the *pPLA-IIγ* is expressed in the reproductive organs [8, 9]. Various studies have also shown how *pPLAs* interact with phytohormones. For example, auxin significantly increases the synthesis of *pPLA-IIδ* and *pPLA-IIIβ*, whereas *pPLA-I* is involved in the biosynthesis of jasmonic acid [10, 11]. *pPLAs* were also involved in regulating the reproductive development of other plants, such as rice and maize [12].

Recently, haploids have been successfully induced using genome sequencing, genetic complementation, and gene editing methods technology of the *MTL* gene, which codes for a pollen-specific phospholipase of the *pPLAs* family [2, 3]. Similarly, haploid seeds have also been obtained by genetic mutations in *Oryza sativa* [13]. In the past years, mutants of *TaPLA* genes in wheat were successfully obtained through gene editing [14]. Furthermore, in addition to monocotyledonous plants, haploid induction has also been done in the dicotyledonous plant (*Arabidopsis*) [15]. These results clearly showed that *pPLAs* have the ability to mediate and even drive haploid development in plants.

Cotton is a significant global crop with substantial commercial value and scientific implications. In cotton, upland cotton is not only the primary source of renewable textile fiber, but also an excellent experimental system for studying polyploidy [16]. The understanding and investigation of *pPLAs* in dicotyledonous plants is primarily limited to *Arabidopsis*, despite the fact that haploids have been induced in a number of monocotyledonous plants. Moreover, the understanding of the development mechanism of *pPLAs* in upland cotton remains largely unknown. In this study, a systematic analysis was conducted on 13 species to investigate their collinearity, domain, 3D structures, expression patterns. Furthermore, the VIGS experiment was conducted in upland cotton to develop *pPLA* silenced plants, which served as a functional confirmation of the *pPLA* gene family. The reasonable analysis and prediction of *GhpPLAs* lay the foundation for further study of *GhpPLAs.*

## Results

### Identification and physiochemical characterization of *pPLAs*

A total of 294 *pPLA* genes were obtained from 13 selected species, including 21 *pPLAs* in *Zea mays* (Zm), 16 in *Oryza sativa* (Os), 23 in *Sorghum bicolor* (Sb), 23 in *Vitis vinifera* (Vv), 17 in *Theobroma cacao* (Tc), 23 in *Glycine max* (Gm), 13 in *Amborella trichopoda* (Atr), 9 in *Selaginella moellendorffii* (Sm), 24 in *G. arboreum* (Ga), 22 in *G. raimondii* (Gr), 46 in *G. hirsutum* (Gh) and 47 in *G. barbadense* (Gb) (Additional file 8: Fig. S1).

The genes were renamed based on their order from front to back on the chromosomes in order to more accurately characterize and identify these *pPLA* genes (Additional file 1: Table S1). A total of 46 *pPLA* genes were identified in upland cotton, and their physicochemical properties were analyzed. All of the 46 genes encoded proteins ranging from 381 (*GhpPLA24* and *GhpPLA45*) to 1329 (*GhpPLA40*) amino acids, with protein PIs varying from 5.07 (*GhpPLA42*) to 9.04 (*GhpPLA12*) and MWs varying from 41.2 (*GhpPLA45*) kDa to 148 (*GhpPLA40)* kDa (Additional file 2: Table S2).

Subcellular localization prediction revealed that most GhpPLA proteins were located in the cytoplasm, with a small amount found in the nucleus and chloroplast (Additional file 2: Table S2). In subgroup I, five genes were located in the nucleus (*GhpPLA27, GhpPLA40, GhpPLA18, GhpPLA14*, and *GhpPLA4*), two in the cytoplasm (*GhpPLA27* and *GhpPLA36*), two in the mitochondria, and one in the chloroplast. In subgroup II, all genes except for *GhpPLA2, GhpPLA12*, and *GhpPLA26* were located in the cytoplasm. Among these three genes, *GhpPLA2* and *GhpPLA26* were found to be simultaneously located in the mitochondria and cytoplasm, while *GhpPLA12* was located in the mitochondria, cytoplasm, and nucleus. In subgroup III-⍺, three genes were located in chloroplasts, *GhpPLA11*, *GhpPLA20* and *GhpPLA29*, and two genes were located in mitochondria (*GhpPLA3*) and cytoplasm (*GhpPLA30*). Four genes in subgroup III-β were localized in both cytoplasm and chloroplast, while the remaining two genes (*GhpPLA15* and *GhpPLA24*) were localized only in the chloroplast. In subgroup III-γ, *GhpPLA23*, *GhpPLA28*, and *GhpPLA44* were located in the cytoplasm, *GhpPLA41* in mitochondria, and *GhpPLA19* in cytoplasm and mitochondria. These results suggested that several *GhpPLAs* are involved in signal transduction processes, such as auxin, pathogens, and inducers, which can activate *pPLAs* [17, 18].

### Evolutionary relationship of *pPLA* in cotton and other 13 plant species

**Fig. 1:**
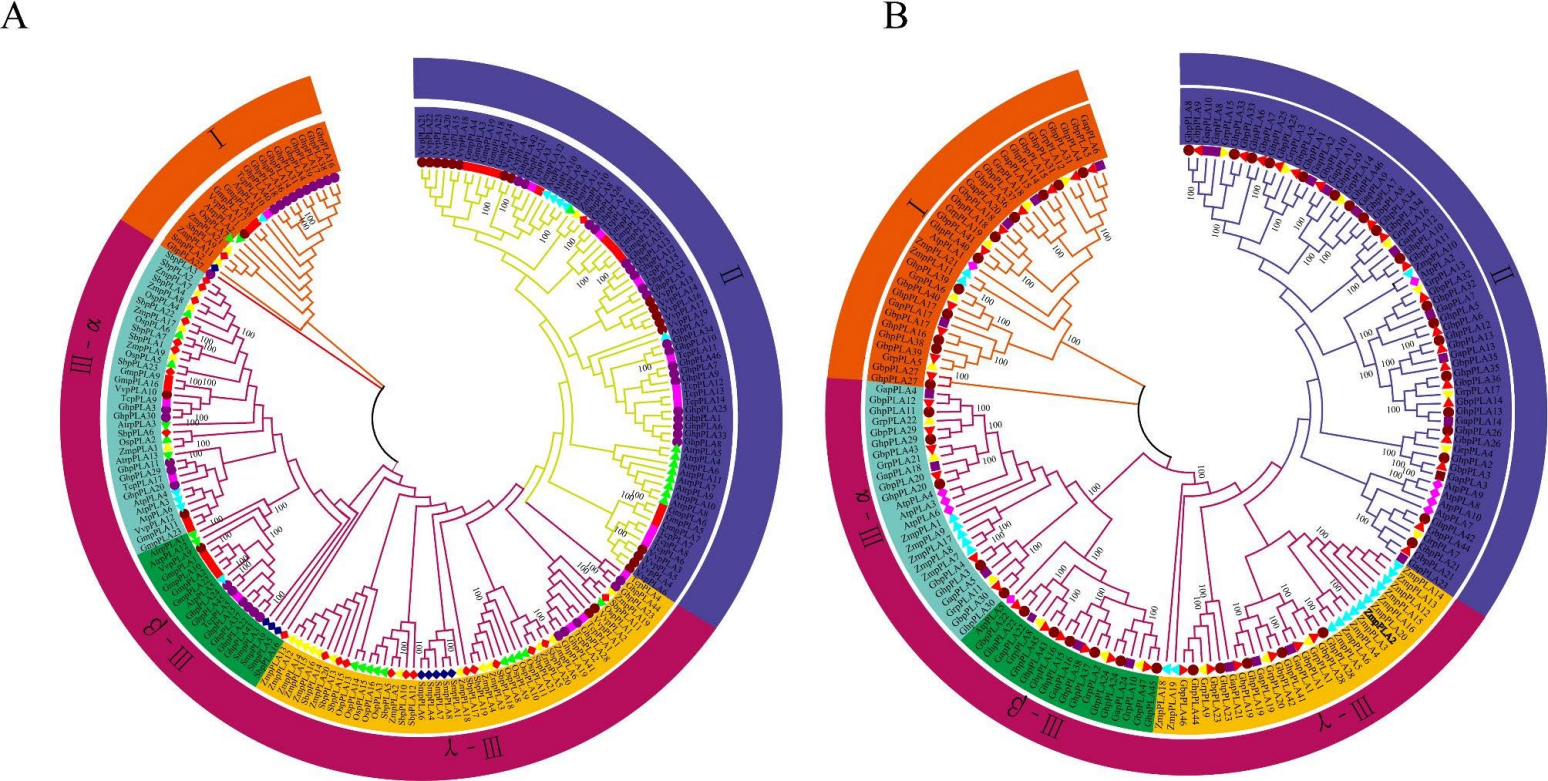
phylogenetic trees of *pPLA* genes. Two unrooted phylogenetic trees were constructed by the MEGA7.0 Maximum-likelihood method. The percentage of replicate trees in which the associated taxa clustered together in the bootstrap test (1000 replicates) is shown next to the branches. All positions with less than 90% site coverage were eliminated. **(A)** Phylogenetic relationship of the 294 identified *pPLA* genes from four cotton species and nine other plant species. **(B)** A phylogenetic tree of *pPLA* genes in four cotton species

According to the clustering of the evolutionary tree and the classification of *AtpPLA*, this evolutionary tree was divided into three subfamilies. Subfamily III was further divided into three subgroups based on the clustering and motif similarity of the evolutionary tree, subgroup (I-III-γ) (Fig. 1A). Among them, *pPLA* genes from dicotyledonous and monocotyledonous plants were distributed in all five classes, in which subgroup III-β having the least number (19 genes) and subgroup II having the greatest number (75 genes). The absence of *pPLA* members from *Amborella trichopoda* in subgroups I and III-γ and *Selaginella moellendorffii* in subgroups II and III-α suggested that these two species may have lost genes during the evolutionary process. *Arabidopsis* has the lowest number, while the two allotetraploid cotton species have the highest numbers in evolutionary tree. The homologous genes of four cotton species are clustered together and closest to dicotyledonous plants (especially *Arabidopsis*), indicating that most of *pPLAs* in cotton is closely related to *Arabidopsis* and other dicotyledonous plants than monocotyledonous plants. As expected, a closer relationship between cotton and cacao than with other species was found in the study, *pPLA* genes in these two species are often phylogenetically linked [19]. Upland cotton has surprisingly undergone tremendous gene family expansion over the evolutionary process, as evidenced by the fact that its gene count in the evolutionary tree is twice or even three times higher than that of other species. These results indicate that gene duplication is the main factor in the evolution and expansion of the *pPLA* gene family in different species.

A phylogenetic tree (Maximum-likelihood; ML) was constructed to demonstrate the evolutionary relationship between diploid *pPLAs* (*G. arboreum* and *G. raimondii*) and allotetraploid cotton *AtpPLAs* (*G. hirsutum* and *G. barbadense*) constructed (Fig. 1B). Allotetraploid species *G. hirsutum* and *G. barbadense* were the most abundant, with ratios close to 1:1 (46 and 47, respectively), whereas diploid species *G. arboreum* and *G. raimondii* were also with ratios close to 1:1 (24 and 22, respectively). Allotetraploid cotton developed by hybridizing diploid cotton during the evolutionary process, as evidenced by the nearly two-fold increase in *pPLA* content in allotetraploid cotton compared to diploid cotton.

### Chromosome mapping and collinearity analysis of *pPLAs*

It was shown by chromosome mapping that 24 *pPLA* genes were mapped on the chromosomes of *G. arboreum*, 22 on *G. raimondii*, and 47 on *G. barbadense*. *G. hirsutum* was slightly different; in addition to the 45 genes located on the chromosomes, one gene was located on the unmapped scaffold (Additional file 9: Fig. S2).

In *G. arboreum*, *pPLAs* were presented on almost every chromosome, except for Chr1, Chr7, and Chr013 (Additional file 9: Fig. S2A). Each chromosome had one to three genes however, Chr7 possessed eight *pPLA* genes, some of which were in tandem and might be the result of duplication occurrences. Comparably, in *G. raimondii*, Chr09 had six *pPLAs*, four of which were in tandem and most likely resulted from duplication events, while Chr01, Chr02, and Chr13 lack *pPLAs*. (Additional file 9: Fig. S2B).

In *G. barbadense*, a total of 47 *GbpPLA* genes were mapped on 19 chromosomes, including A2, A3, A5, A6, A8, A9, A010, A011, A012, D2, D3, D4, D5, D6, D8, D9, D010, D011 and D012 (Additional file 9: Fig. S2C). In *G. hirsutum*, *pPLA* genes were located on 18 chromosomes, including A03, A05, A06, A08, A09, A10, A11, A12, D02, D03, D04, D05, D06, D08, D09, D10, D11, and D12. Another gene (*GhpPLA46*) was located on an unmapped scaffold (Additional file 9: Fig. S2D). These findings indicated that the evolution of *GhpPLAs* was relatively mature. However, the number of genes on most chromosomes was observed to be dissimilar, indicated that *pPLAs* were unevenly and randomly distributed across the chromosomes of *G. barbadense* and *G. hirsutum*. This uneven distribution might have been caused by gene loss during the evolutionary process or incomplete genome assembly. Not only that, the number of *pPLAs* on At and Dt was close to 1:1, and the number and distribution of *pPLAs* on homologous chromosomes of different subgenomes were highly similar in *G. hirsutum* and *G. barbadense*. These results suggested that *pPLA* genes were relatively conserved in upland cotton and island cotton genomes. Notably, two tetraploid cotton sub-genomes, A and D, differed significantly from diploid cotton genomes in terms of both the number and the distribution of genes on their chromosomes. These findings imply that *pPLAs* might have experienced chromosomal rearrangement during cotton’s evolutionary history.

**Fig. 2:**
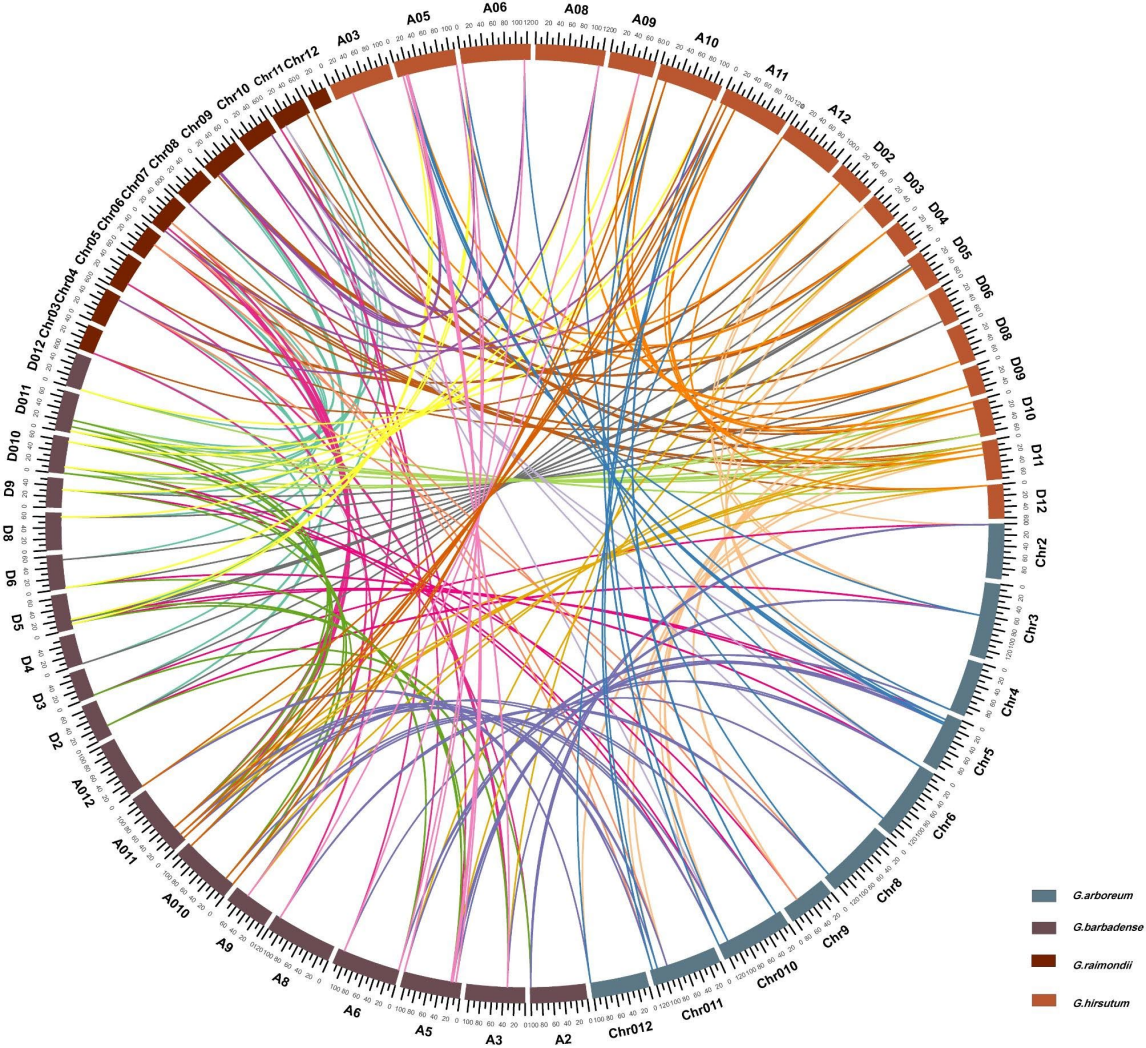
The collinearity relationships of *pPLA* genes of four cotton species. The chromosomes of *G. arboreum, G. raimondii, G. hirsutum*, and *G. barbadense* were shown with little blue, kermesinus, orange, and brown colors, respectively

**Fig. 3:**
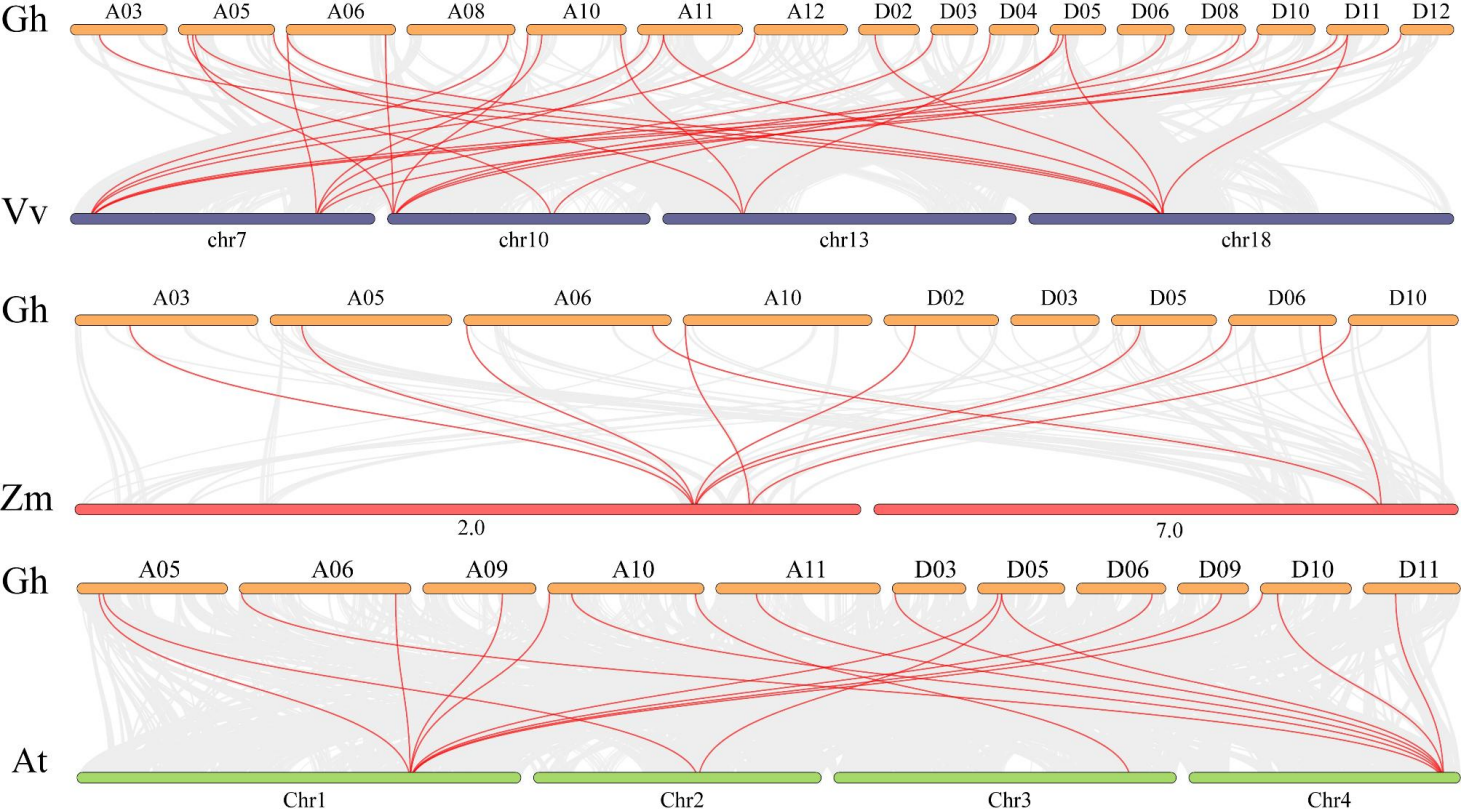
The synteny analysis of *pPLA* genes between *G. hirsutum* and three representative plant species. The blue lines highlight the syntenic *pPLA* gene pairs. The specie names with the prefixes “Gh”, “Vv”, “Zm” and “At” indicate *G. hirsutum*, *Vitis vinifera*, *Zea mays*, and *Arabidopsis thaliana*, respectively

In the process of plant evolution, large fragment repetition of chromosomal regions was followed by gene loss, small-scale repetition, chromosome rearrangement, and other processes, which eventually led to the complexity of plant genomes [20]. Gene duplication mainly includes whole genome duplication (WGD), fragment duplication, and tandem duplication, which provides new genetic material for evolution and generates new gene functions, which is also an important reason for the widespread existence of gene families [21–23]. A total of 222 orthologous/paralogous gene pairs were obtained by collinearity analysis of four cotton species (Fig. 2; Additional file 3: Table S3). Most of these gene pairs experienced whole genome duplication (WGD), and most of them did not have tandem duplication, indicating that the amplification of cotton *pPLA*s mainly depended on WGD in the evolution process. These collinear pairs indicated that the evolution of cotton *pPLA*s is conserved. To further investigate the evolution and developmental mechanism of *GhpPLA*s, homo-linear analysis was performed on upland cotton and three representative species, including one dicotyledon (*Arabidopsis thaliana*) and two monocotyledons (*Vitis vinifer* and *Zea mays*) (Fig. 3). The results showed that there were 18 orthologous pairs in upland cotton and *Arabidopsis thaliana*, and a total of 10 and 29 *pPLA*s showed syntenic relationships with *Vitis vinifer* and *Zea mays*, respectively. This suggests that the homology of upland cotton to *Vitis vinifer* is higher than *Arabidopsis thaliana*, but lower than maize. Moreover, *GhpPLAs* also underwent additional genome-wide duplication due to the fact that there were 10 or more orthologous pairs between upland cotton and each species. Some *GhpPLAs* were colinear with *Arabidopsis thaliana*, maize and grape, suggested that they may play an important role in evolution. The expression patterns of orthologous pairs were similar, so *pPLAs* in upland cotton might have similar effects with *pPLA*s in different species.

### Conserved motif and structural analysis of *GhpPLAs*

**Fig. 4:**
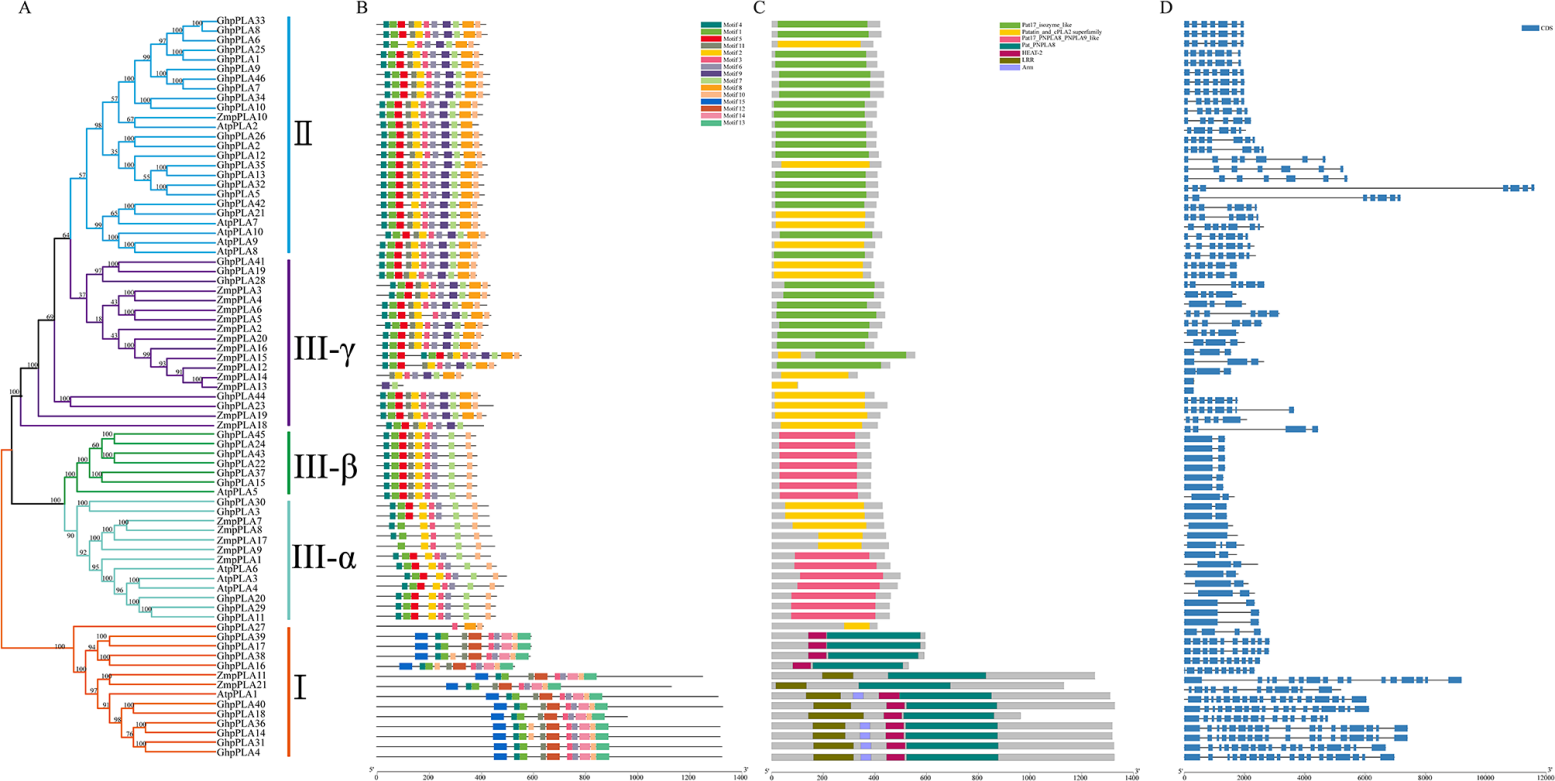
The gene structure, conserved motifs and domains in *pPLA* genes between *Arabidopsis* and four cotton species. **(A)** The ML phylogenetic tree was constructed based on the full-length sequences of *Arabidopsis*, Maize and four cotton species pPLA proteins using MEGA 7.0 software. Different subfamilies are highlighted in different colors. **(B)** Conserved motif of *Arabidopsis*, Maize and four cotton species. Different colored squares represent different motifs, named motif1-motif15. **(C)** The conserved domains in *Arabidopsis*, maize and four cotton species pPLA proteins. **(D)** The exon-intron structure of *Arabidopsis*, maize, and four cotton species *pPLA* genes. Blue for exons; black lines for introns

Conserved domains, motifs, and gene structure analysis were carried out, as these are directly linked with the function of a gene family (Fig. 4). Structural analysis demonstrated that different subgroups contained different numbers of introns and exons. Each gene in subgroup I contained more than 10 exons and more than 9 introns, and upland cotton genes (*GhpPLA18*, *GhpPLA36*, *GhpPLA14*, *GhpPLA31* and *GhpPLA4*) had the highest number of exon and intron, with a total of 18 exons and 17 introns. Whereas, subgroup III-α contained only two exons and one intron, and subgroup III-γ contained up to six exons. Subgroup II basically contained 7 exons and 6 introns (Fig. 4D). The number of exons in different subfamilies varied, but the number of exons in the same subfamily was almost identical and had the same gene structure. This fully indicted that genes from different subfamilies have different biological functions, while genes from the same subfamily have a certain degree of conservation.

**Fig. 5:**
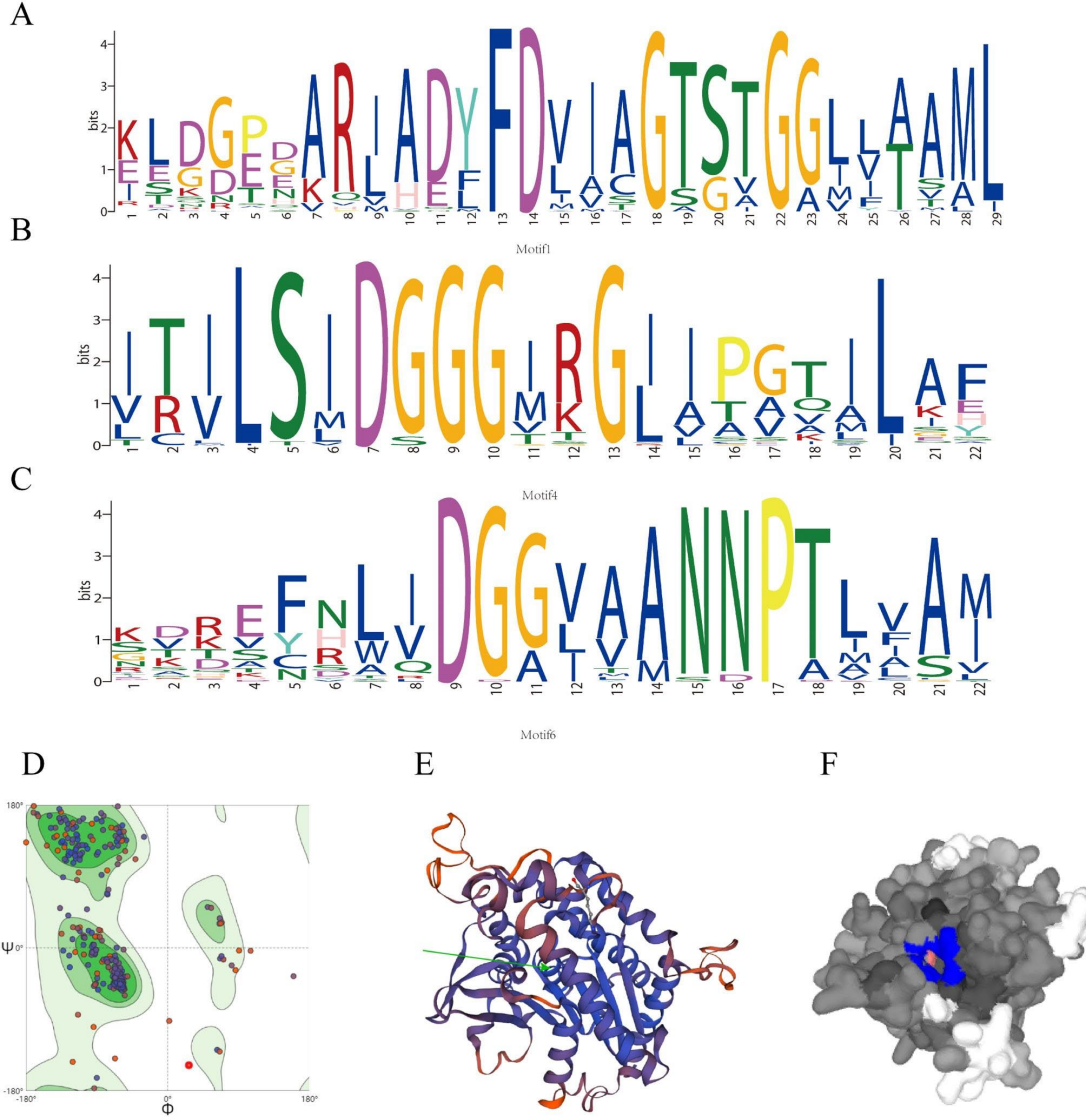
Sequences logos of conserved motifs and 3D structure of GhpPLA proteins. **(A)** Motif 1. **(B)** Motif 4. **(C)** Motif 6. **(D)** Ramachandran plot for predicting 3D structure of GhpPLA proteins. **(E)** Prediction of the 3D structure of GhpPLA proteins. The green arrow represents the active site. **(F)** Prediction of binding sites of GhpPLA proteins ligand. The blue areas represent ligand binding sites

A total of 15 conserved motifs (named as Motif 1-Motif 15) were predicted in upland cotton (Fig. 4B), among which subgroup I contained 10 conserved motifs, Subgroup III-α contained 8 conserved motifs, and subgroup III-γ contained 9 conserved motifs. Subgroup III-γ and subgroup II had the largest number of conservative motifs, with a total of 11. The pPLA proteins of cotton, *Arabidopsis* and maize all contained a conserved ‘GXSXG’ typical serine hydrolase motif (named Motif 1) that catalyzes the dimer of serine and aspartic acid (Fig. 5A). Apart from ‘GXSXG’, all contained a conserved ‘DGG’ motif (named Motif 6) which was known as an enzyme active site and it was localized in the first one of the five Ca^2+^ binding β-rolls that formed the so-called C-terminal repeat domain [24] (Fig. 5C). However, in addition to subgroup I, the other subgroups also contained the ‘DGGGXRG’ motif (named Motif4), which were binding sites for phosphate anion element (Fig. 5B).

### Homology modeling of ertiary 3D protein structure of GhpPLA

The evaluation of the projected 3D configurations was conducted by assessing their GMQE score and the highest percentage of amino acids in the favored area of the Ramachandran plot. Among the templates considered, 1OXW.1B was deemed the most appropriate due to its substantial sequence similarity (43%) and GMQE score (0.72) with the query proteins. The target sequence had a length of 396 residues, while the template had a coverage range of 16–392 residues. More than 90% of amino acid residues were present in the Ramachandran plot (Fig. 5D), which can be considered as a high-quality protein model [25]. As depicted in Fig. 7B, the structure model of GhpPLAs revealed that they were primarily comprised of α-helices with a minor number of β-sheets. These proteins exhibited an α/β class protein fold with around three layers, as described in previous research [26]. Additionally, the catalytic Ser-Asp dyad was presented, with the serine residue located within the highly conserved lipase motif (GXSXG) [27]. Its folded topology and active site structure were closely related to the catalytic domain structure of human cPLA2α [28, 29]. By importing the PDB file into Prankweb, five ligand binding sites were predicted, two of which were more conservative. Based on the sequence analysis of each ligand binding site, pocket1 was identified as the true ligand binding site with a ser-catalyzed residue (Fig. 5F).

### Promoter *cis*-element analysis of *GhpPLAs*

**Fig. 6:**
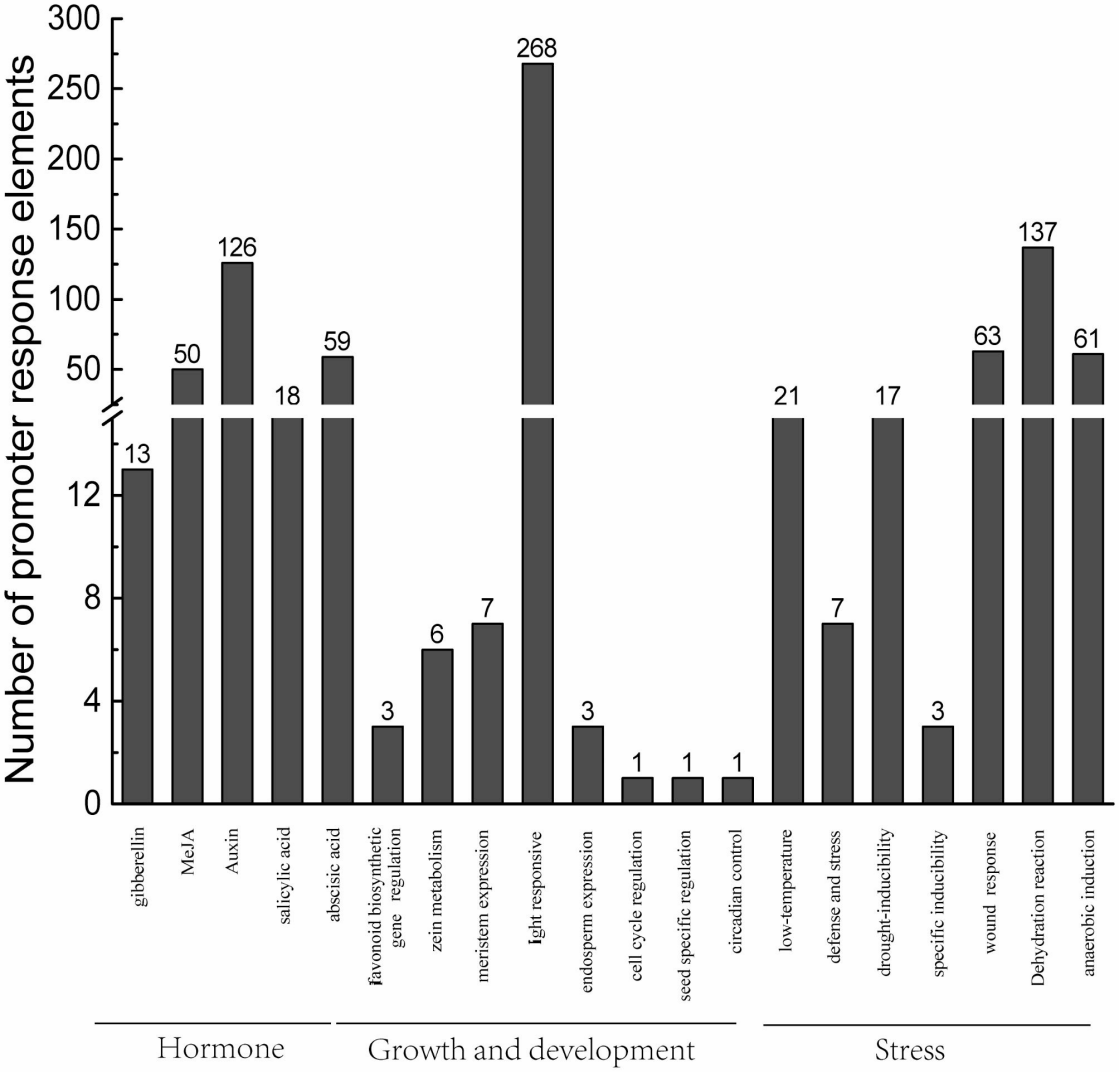
The number statistics of *GhpPLAs cis-*regulating elements in the promoter

**Fig. 7:**
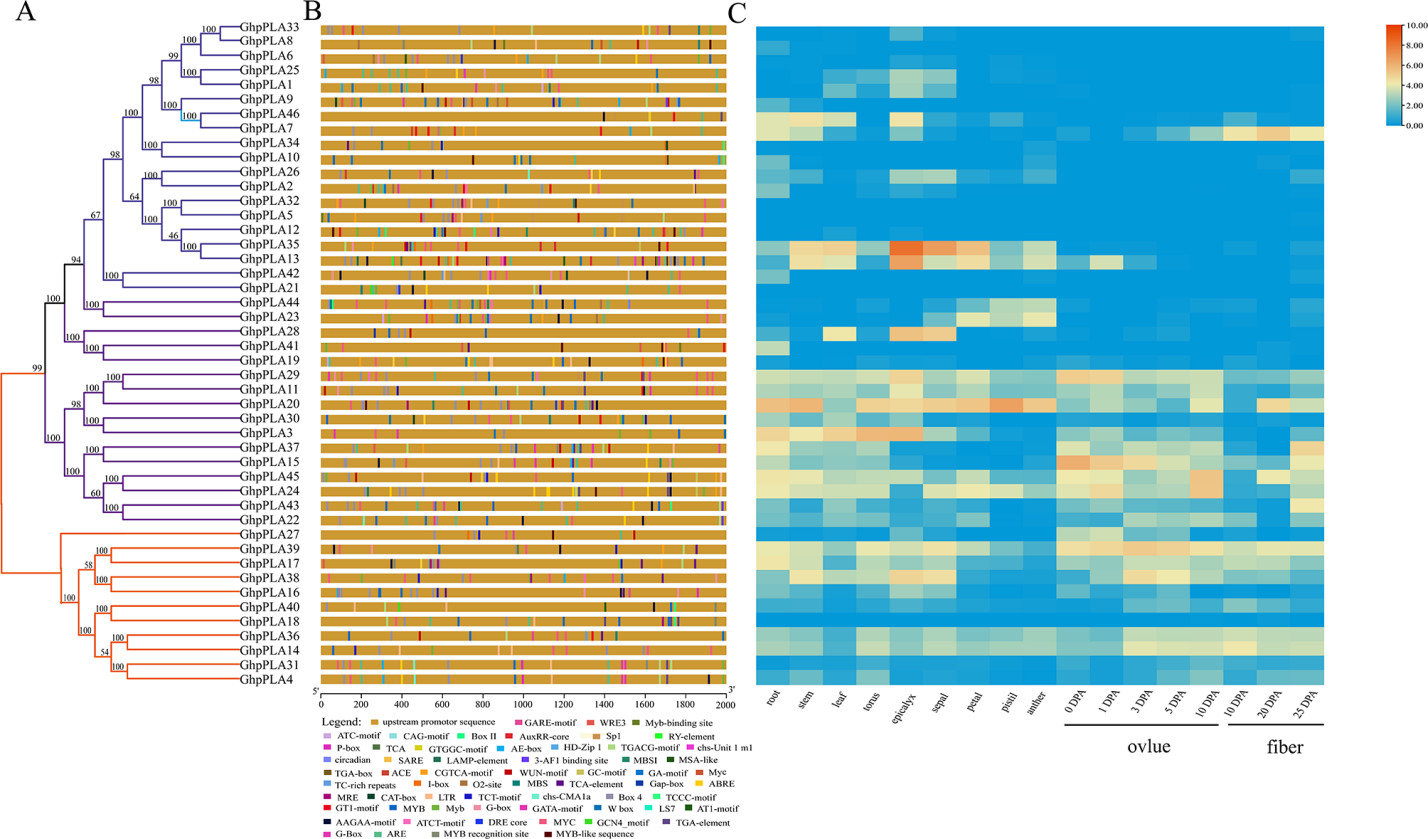
Analysis of *GhpPLA* genes promoter and its expression pattern in different tissues. **(A)** Phylogenetic tree of *GhpPLAs*. **(B)***Cis*-elements in promoters of *GhpPLAs*. **(C)** Expression pattern of *GhpPLAs* in different tissues

A *cis*-regulating element refers to a sequence within a gene sequence that plays a role in regulating gene expression. These elements can participate in the transcriptional regulation of a dynamic network of gene activities that control various biological processes [30]. In the case of *GhpPLA*, a total of 61 *cis-*regulating elements were identified, which were classified into three categories: hormone-acting elements, stress-acting elements, and growth and development-acting elements (as shown in Figs. 6 and 7, and detailed in Additional file 4: Table S4).

The *pPLA* genes of upland cotton contained 266 hormone *cis-*regulating elements, 29 growth *cis-*regulating elements and 309 abiotic stress *cis-*regulating elements (Figs. 6 and 7B; Additional file 4: Table S4). Auxin-related components (47.37%) were the most prevalent among phytohormone-related action elements. MYB, MYB-like sequence, TGA-element, AuxRR-core and MYB recognition site. Salicylic acid responsiveness elements (TCA-element and SARE), gibberellin-responsive elements (P-box and GARE-motif), MeJA-responsiveness elements (CGTCA-motif and TGACG-motif) and abscisic acid responsiveness elements (ABRE and AAGAA-motif) were counted as 6.77%, 4.89%, 18.8% and 22.18%, respectively. Conclusionary, there were more auxin reaction elements and abscisic acid reaction elements, indicated that most *pPLA* genes might be involved in the reproductive development and the senescence process of plants.

The growth and development-acting elements had the highest number of light-responsive elements, which made up approximately 92.41% of the total number of identified *cis*-acting elements (Figs. 6 and 7B; Additional file 4: Table S4). ATC-motif, GATA-motif, G-box, AE-box, GT1-motif, ATCT-motif, GATA-motif, GA-motif, Box4, chs-CMA1a, MRE, Sp1, ACE, 3-AF1 binding site, I-box, TCCC-motif, LAMP-element and Box II all belong to the light-responsive elements. It was hypothesized that the expression of *GhpPLAs* might be regulated by light. Additionally, specific response elements such as CAT-box, GCN4-motif, MSA-like, O_2_-site, RY-element, and circadian were also identified in relatively small numbers. For instance, there was only one circadian response element and one RY-element present in *GhpPLAs*. The MSA-like response element was also limited to only one, and they were only located in *GhpPLA44, GhpPLA13*, and *GhpPLA2*. Based on these findings, it can be speculated that *GhpPLA44* might be regulated by day-night alternation, while *GhpPLA13* might be specifically regulated in seeds, and *GhpPLA2* by the cell cycle.

The most biotic or abiotic stress elements were dehydration-responsive elements, accounting for 44.34% of the total, with a total of 137 elements, such as Myb, MYC, Myb-binding site and DRE core (Figs. 6 and 7B; Additional file 4: Table S4). Secondly, the wound reactive element (W-box, WRE3, WUN-motif) and anaerobic inducible element including ARE were 63 and 61 respectively, which were close to 1:1. The reactivity element involved in low temperature responsiveness was LTR. Some elements can be involved in drought induction, for example, MBS. The element involved in defense and stress response were TC-rich repeats. The least number of anoxic-specific inducibility elements were GC-motif. The expression of *GhpPLA* genes might be primarily influenced by light, auxin, and dehydration responses, and they might play a role in controlling the process of upland cotton reproductive development, according to predictions derived from the investigation of these reaction elements.

### Expression patterns of *GhpPLA* and qRT-PCR analysis

The heat map of the *GhpPLA* expression level was constructed using *GhpPLA* gene expression data that was available on the RNA-seq database (Fig. 7C; Additional file 5: Table S5). Heat map clearly showed that only *GhpPLA7* was specifically expressed in cotton fiber, which might be important for the growth of cotton fiber. In addition, *GhpPLA23* and *GhpPLA44* were specifically expressed in anthers, pistils, and petals, which were probably related to the reproductive development of cotton. The remaining genes were expressed in vegetative organs and reproductive organs, or only specifically expressed in vegetative organs. The previous phylogenetic tree results suggested that the induced haploid gene *GRMZM2G471240* (renamed *ZmpPLA2*) in maize belongs to subgroup III- γ. Five upland cotton genes (*GhpPLA23*, *GhpPLA44, GhpPLA41, GhpPLA19*, and *GhpPLA28*) with the closest genetic relationship to this gene and a gene *GhpPLA7* specifically expressed in fibers were selected for qRT-PCR analysis.

**Fig. 8:**
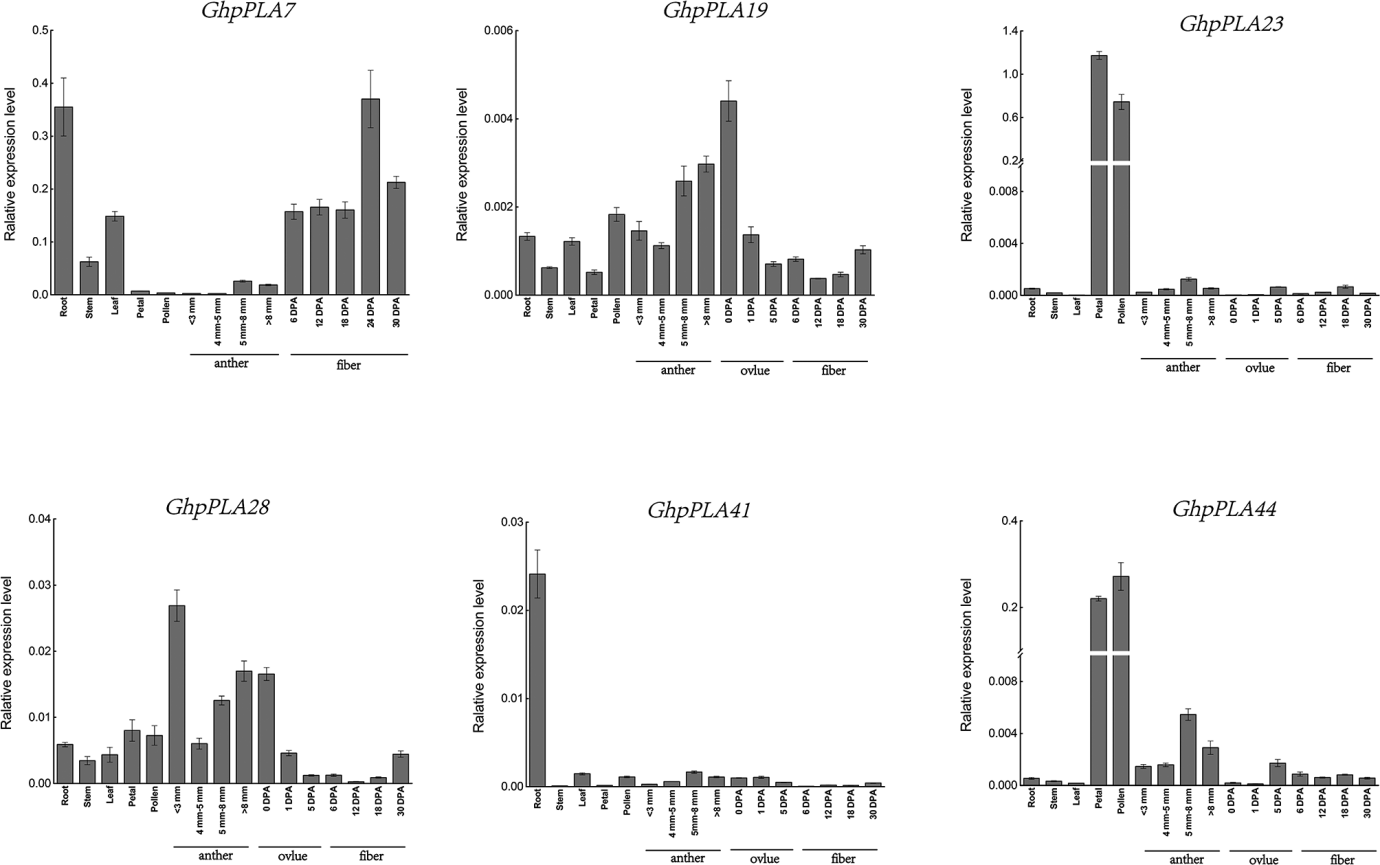
The expression of *pPLAs* in different tissues in *G. hirsutum*

Expression analysis elucidated that *GhpPLA7* was expressed exclusively in roots and fibers, with a particularly high expression in roots and 24 DPA fibers (Fig. 8, Additional file 6: Table S6). This observation suggested that *GhpPLA7* might play a crucial role in regulating the development of cotton fibers. It was shown in Fig. 8 that the expression level of *GhpPLA19* in ovules at 0 DPA was slightly higher than other tissues, but the database showed that the expression level of each tissue was not high, which might be due to the selection of *G. hirsutum* cultivar Zhongmiansuo 100 and the selection of *G. hirsutum* cultivar TM-1 in the database, so the findings were little inconsistent. Similarly, in the public database, *GhpPLA28* only expressed in leaves, while qRT-PCR analysis showed that *GhpPLA28* was also expressed in anthers. This might be due to subtle differences between different cotton varieties, but it has no impact on later analysis and experimental research. Among them, *GhpPLA41* was specifically expressed in the roots, and this gene might not affect the reproductive and developmental processes of cotton. Furthermore, expression analysis showed that *GhpPLA23* and *GhpPLA44* were specifically expressed in pollen and petals on the day of flowering, and these two genes might be the breakthrough in inducing haploid production.

### Development of *GhpPLA23* and *GhpPLA44* silent plants via VIGS

**Fig. 9:**
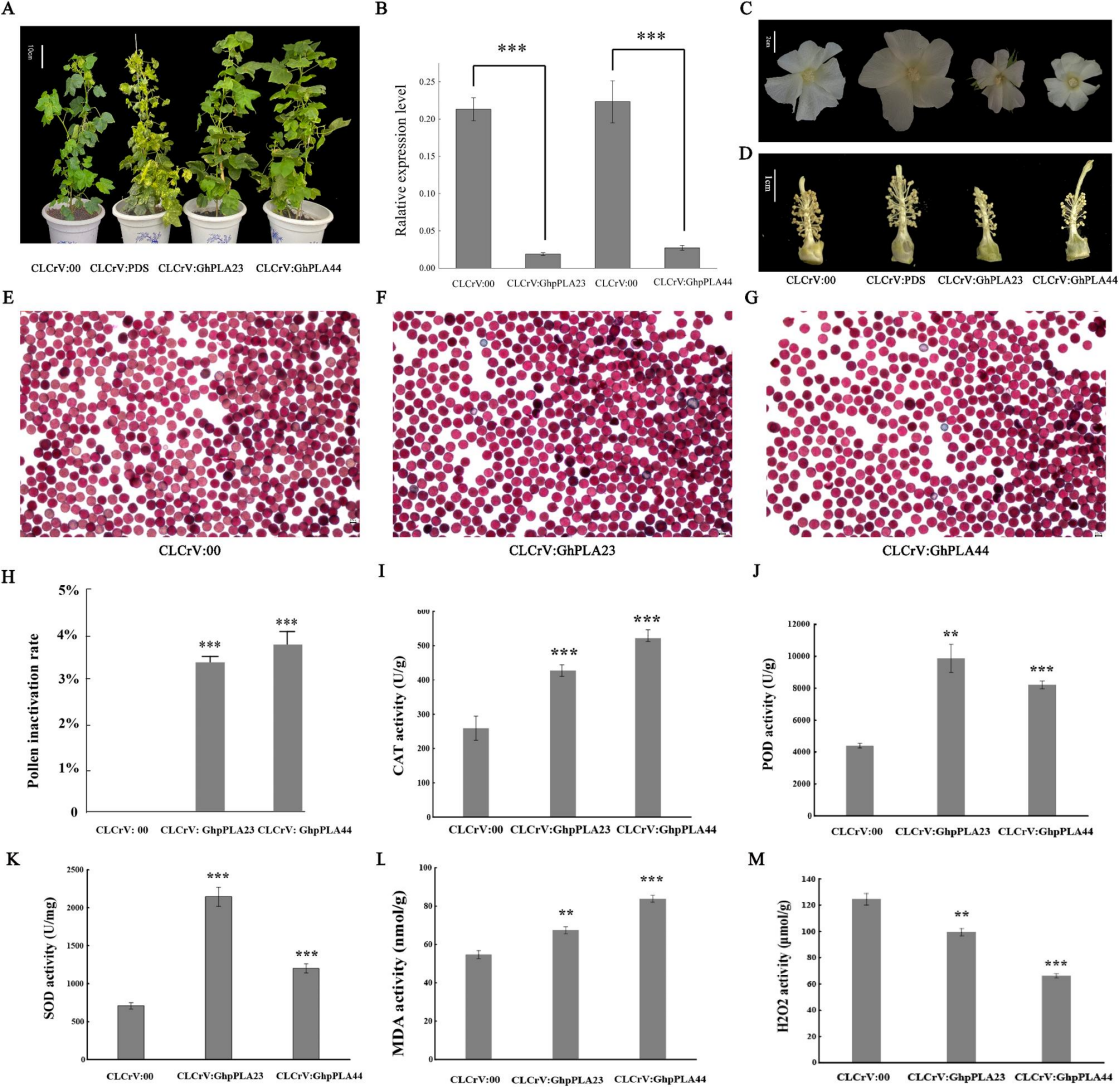
Phenotypic observation and changes of pollen viability in WT plants and plants with VIGS vectors. **(A)** Height comparison of VIGS vector containing plants. **(B)** Changes of pollen expression after VIGS. **(C)** Comparison of flower size difference. **(D)** Comparison of stigma size difference. **(E)** Pollen vitality of CLCrV: 00. **(F)** Pollen vitality of CLCrV: GhpPLA23. **(G)** Pollen vitality of CLCrV: GhpPLA44. **(H)** Pollen non-viable rate in plants with VIGS vectors. **(I)** Changes of CAT activity in pollen of plants with VIGS vectors. **(G)** Changes of POD activity in pollen of plants VIGS vectors. **(K)** Changes of SOD activity in pollen of plants with VIGS vectors. **(L)** Changes of MDA activity in pollen of plants with VIGS vectors. **(M)** Changes of H_2_O_2_ activity in pollen of plants with VIGS vectors. *** represent significant difference at the level of *p* < 0.001. ** represent significant difference at the level of *p* < 0.01

Based on the analysis of *cis*-acting elements and expression patterns of *GhpPLA*, it was speculated that *GhpPLA* gene family might have potential functions in cotton reproductive development. Therefore, *GhpPLA23* and *GhpPLA44* were selected as candidate genes for further functional studies to prove this inference. The gene-silenced cotton plants were grown in a greenhouse until maturity, the plant heights of the positive plants carrying CLCrV: PDS, the negative control plants, and the two plants with genes silencing were basically the same, indicating that *GhpPLA23* and *GhpPLA44* didn’t affected cotton plant height after being silenced (Fig. 9A). However, it was observed that the petals and stigmas were obviously smaller (Fig. 9C and D). In addition, it was detected that the silencing of these two genes not only led to a significant reduction in the number of anthers but also caused a substantial decrease in the amount of released pollen (Fig. 9C). These findings suggested that the silencing of *GhpPLA23* and *GhpPLA44* has a considerable impact on the development of cotton floral organs, particularly on anthers and pollen, which will ultimately affect the reproductive developmental process of upland cotton. The qRT-PCR results demonstrated that the yield of pollen in plants subjected to VIGS was substantially lower compared to the control plants (Fig. 9B). All these findings indicated the significant impact of *GhpPLA23* and *GhpPLA44* on pollen development, thereby provided further evidence for their role in the reproductive development of upland cotton.

### Effect of *GhpPLA23* *GhpPLA44* and silencing on pollen activity and physiological index

The control plant group showed almost 100% pollen viability with only one or two inactive pollen grains, while the VIGS plants exhibited a decrease of approximately 3.6% in pollen viability, indicating a significant decline trend (Fig. 9E, F, G and H, Additional file 7: Table S7). This observation also indicated, *GhpPLA23* and *GhpPLA44* might have an impact on pollen viability and development that indirectly affects the pollen fertilization. By detecting POD, SOD, CAT, H_2_O_2_, and MDA in pollen, it was observed that level of CAT, POD, SOD, and MDA in pollen increased dramatically in the silenced plants, although H_2_O_2_ showed a negative trend (Fig. 9). In maize, it has been confirmed that knocking out *ZmPLA1* lead to the ROS outburst, which lead to DNA damage in sperm cells and cause induction of haploids containing only the maternal genome. Therefore, the significant changes in ROS and ROS scavengers in cotton after gene silencing of *GhpPLA23* and *GhpPLA44* may also be caused by the outbreak of ROS after gene silencing, which is consistent with research in maize [31]. These two genes play an important role in sperm cell development and fertilization in upland cotton, but respective mechanisms need further exploration and verification.

### Interaction network of GhpPLA proteins

**Fig. 10:**
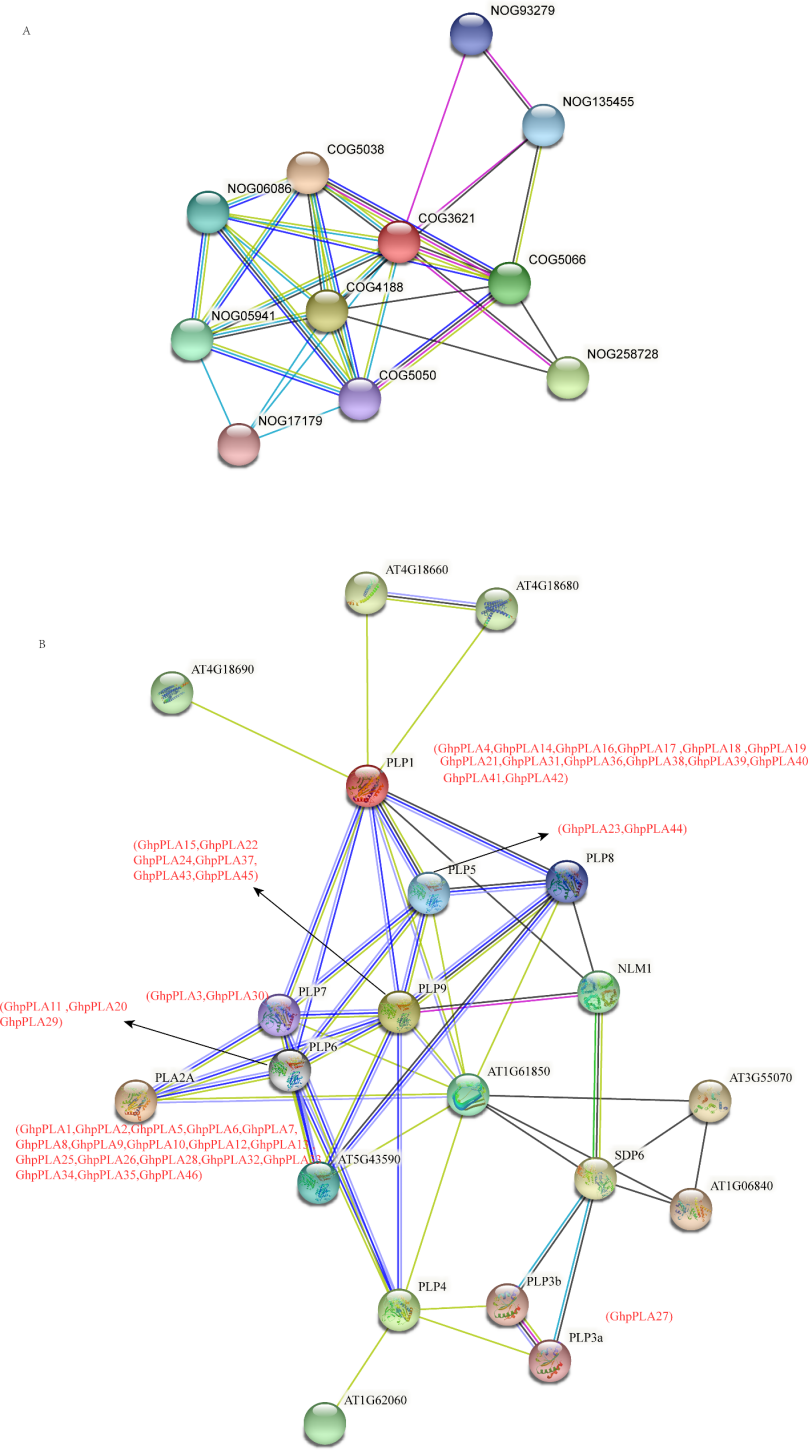
Interaction network of pPLA proteins. **(A)** Interaction network of pPLA proteins families. **(B)** Interaction network of GhpPLA proteins with other proteins. The red letters represent GhpPLA proteins

The protein-protein interaction network is composed of individual proteins through interactions with each other [32]. To gain insight into the function of pPLA proteins, STRING data was used to construct and analyze protein interaction network based on *Arabidopsis* homologous sequences using protein family and multi-sequence search (Fig. 10). Through the in-depth study of *Arabidopsis*, the function of GhpPLA proteins can be speculated. By searching the protein families (Fig. 10A), Patatin-like phospholipase/acyl hydrolase (COG3621) was found in the center, and other pathways, such as diacylglycerol choline phosphotransferase activity (COG5050) [33], which can reduce seed metabolism. The pathway of FFAT motifs (COG5066) [34] targets cytoplasmic proteins to the surface of the endoplasmic reticulum as well as the nuclear membrane, suggested GhpPLAs might be involved in some signal transduction processes. Ca^2+^ dependent lipid-binding protein (COG5038) was found around the interaction network, suggested that some GhpPLAs also participate in pollen germination and pollen tube growth through this signaling pathway [35–37].

By the method of multiple sequences search (Fig. 10B), the interaction between GhpPLAs was fully demonstrated. The results showed that PLP8, PLP9 (GhpPLA45, GhpPLA43, GhpPLA37, GhpPLA24, GhpPLA22, GhpPLA1*5*) and PLP1 (GhpPLA4, GhpPLA14, GhpPLA16, GhpPLA17, GhpPLA18, GhpPLA19, GhpPLA21, GhpPLA31, GhpPLA36, GhpPLA38, GhpPLA39, GhpPLA40, GhpPLA41, GhpPLA42) directly interacted with NLM1, suggested that the expression levels of PLP8 and PLP9 might be decreased due to ABA, NaCl and drought stress induction. Interaction of PLP3 (GhpPLA27) with SDP6 indicated that PLP3 was involved in storage lipid catabolism.

## Discussion

Patatins refer to proteins found in potato tubers that possess acyl hydrolysis activity [38]. The patatin catalytic domain is present in various organisms including plants, animals, bacteria, etc., and can effectively catalyze the hydrolysis of both phospholipid sn-1 and sn-2, thereby releasing free fatty acids simultaneously. According to the genome-wide identification of 13 species, a total of 294 *pPLA* genes were identified and classified into three distinct subfamilies. Notably, allotetraploid cotton had the greatest number of *pPLA* genes compared to the other studied species. Previous studies have identified 10 *pPLA* genes in upland cotton [39]. However, this current study has conducted a more comprehensive analysis and identified a total of 46 *pPLA* genes, including those identified in previous studies. Moreover, the study has also verified the functions of candidate genes in reproductive development. Out of the 46 *pPLA* genes identified in upland cotton, 24 genes were in At genome, 22 genes in the Dt genome, and one gene was located on the unmapped stent, indicating that the evolution of the *pPLA* gene family was relatively conservative and mature in upland cotton. Compared to other species and diploid cotton, upland cotton has 2–3 times more *pPLA* genes, which might be attributed to chromosome doubling and genome amplification in allotetraploid cotton. According to a genome-wide duplication investigation, *G. arboreum* and *G. raimondii* underwent genome-wide duplication specific to cotton 1.6 million years ago. Another genome-wide duplication occurred between 13 and 20 million years ago, and upland cotton originated between 1 and 2 million years ago [40–43]. The complexity and functional expansion of cotton *pPLAs* were mainly caused by gene duplication. During the evolution of *GhpPLAs*, random distribution and uneven number of *GhpPLAs* on chromosomes were caused by gene loss, gene increase, chromosome rearrangement, and incomplete genome assembly.

*pPLAs* have a specific patatin domain, mainly PLP1, PLP2, PLP3, PLP5, PLP6, PLP7 and PLP9 in upland cotton. The conserved motif analysis showed that all upland cotton contained a conserved ‘GXSXG’ typical serine hydrolase motif [27] and a conserved ‘DGG’ motif, in which ser residue in ‘GXSXG’ was the catalytic central residue, which catalyze the dimer of serine and aspartic acid. All these results indicated the evolutionary conservation of *GhpPLAs.* The gene structure of *GhpPLAs* showed that the number of exons were different in different subfamilies and the same subfamily had a similar gene structure, indicated that the biological functions of different subfamilies might differ, and the biological functions of the same subfamily were likely to be similar. The similarities and differences in *GhpPLAs* domains, conserved motifs, and gene structures might be related to conserved and sub-functionalization, mainly due to gene duplication during evolution [43]. Subcellular localization predicts that *GhpPLAs* are mainly located in the cytoplasm, which may be involved in plant signal transduction, such as auxin, pathogens, and other inducers [17, 18]. Moreover, through the analysis of homeopathic elements in the upstream 2000 bp region of the promoter, it has shown that *pPLAs* maybe responsive to auxin, abscisic acid, light, and drought, indicating that these elements are important for *pPLA* gene regulation. It was speculated that *GhpPLAs* may regulate the reproductive development process of upland cotton through the effect of auxin. The protein interaction network showed that pPLA proteins acted directly with diacylglycerol choline phosphotransferase activity (COG5050) [33], FFAT motifs (COG5066) [34], and Ca^2+^ dependent lipid-binding protein (COG5038) [35–37], suggested that *pPLAs* might be involved in seed lipid storage and plant signal transduction. Moreover, it was reported that Ca^2+^ dependent lipid-binding protein was involved in pollen germination and pollen tube growth [36], so *GhpPLAs* might play a role in pollen and thus affect the reproductive development of upland cotton.

Silencing of *GhpPLA23* and *GhpPLA44* in upland cotton has been shown to have a significant effect. It has observed that silencing of *GhpPLA23* and *GhpPLA44* caused the smaller the petals, stigmas, and anthers. And the amount of viable pollen has also been significantly reduced. This further supports the hypothesis that *GhpPLAs* play a crucial role in the reproductive development of upland cotton. In addition, the expression of pollen has a significant decreased trend compared with the control. Through the observation of the pollen vitality in plants after VIGS, it has shown that there are certain level of non-viable pollen. All of the evidence points to the possible role of *GhpPLA23* and *GhpPLA44* during upland cotton’s reproductive development. In addition, ROS has been were greatly impacted in the *GhpPLA23* and *GhpPLA44* silenced plants. The pollen’s POD, H_2_O_2_, CAT, MDA, and SOD levels all differed dramatically in *GhpPLA23* and *GhpPLA44* silenced plants compared with control [31]. Moreover, studies have proved that haploid induced by *pPLA* were because of ROS explosion, and the sperm cell DNA rupture finally produced the haploid containing only the maternal genome [44]. Therefore, *GhpPLA23* and *GhpPLA44* are likely to be the key genes for the production of haploid for upland cotton.

Haploid breeding plays an important role in crop breeding. Previous studies have found some candidate genes responsible for the haploid, such as *CENH3* [45, 46], *DMP* [47–49] and *PLD3* [50]. *MATL*, also known as *ZmPLA1* (renamed *ZmpPLA2* in this article) belongs to the *pPLA* gene family and can induce maize haploid [11, 12]. In recent years, researchers have successfully developed rice haploids by knocking out *MATL* homologous genes in rice and wheat, indicating that *pPLA* gene mutations is linked with the haploid induction in monocotyledons [13, 14]. Additionally, *pPLAs* are also involved in plant growth and development regulation in *Arabidopsis thaliana* [15]. In this study, several genes closely related to *ZmPLA1* were analyzed systematically. It was found that *GhpPLA23* and *GhpPLA44* were specifically expressed in pollen and petals, and the silencing of these two genes significantly affected the size of cotton petals and stigma, the number and expression of pollen and various physiological indicators. It is hypothesized that, *GhpPLA23* and *GhpPLA44*, could serve as potential candidate genes for future haploid breeding of cotton. However, it is important to note that whether these genes can induce haploids requires further investigation. Currently, this study confirmed that *GhpPLAs* play a role in the reproductive development of upland cotton, and further research is needed to explore their potential applications in haploid breeding.

## Conclusions

In this study identification and phylogenetic analysis of cotton, as well as, the analysis of the chromosome location, conservative motif, gene replication events, protein structure, and interaction network of 46 genes in upland cotton were carried out to explore the role of *pPLAs* in haploid induction. These genes were classified into three subgroups, leading to a deeper understanding of *pPLA*. The expression analysis of *GhpPLA23* and *GhpPLA44* and the results of VIGS proved that these two genes play an important role in the reproductive development of upland cotton, which laid a foundation for the study of *pPLAs* in the reproductive process of upland cotton and the induction of haploid.

## Materials and methods

### Databases

The genome information of four cotton species, including *G. arboreum* (ZJU, version 1.0), *G. raimondii* (ZJU, version 2.0), *G. hirsutum* (ZJU, version 1.0) and *G. barbadense* (ZJU, version 1.0), were downloaded from CottonFGD (http://cottonfgd.org) [51]. The genome information of other nine species, including *Arabidopsis thaliana* (TAIR, version 10), *Zea mays* (Ensembl-18_2010_01), *Amborella trichopoda* (version 1.0), *Glycine max* (version 1.0), *Oryza sativa* (version 7.0), *Sorghum bicolor* (version 3.1.1), *Selaginella moellendorffii* (version 1.0), *Theobroma cacao* (version 2.1) and *Vitis vinifera* (version 2.1), were acquired from phytozome (https://phytozome-next.jgi.doe.gov/ accessed on 22 November 2020) [52].

### Identification of pPLA family members

The genome annotation data (in gff3 format) and gene sequence data (in Fasta format) for four cotton species were downloaded and converted to protein information using TBtools [53]. The amino acid sequences of AtpPLAs were used as query sequences to search for the *pPLA* genes in four cotton protein databases using the blast program. Subsequently, Pfam (http://pfam.xfam.org/) [54] and SMART (http://smart.embl-heidelberg.de/) [55] were used to screen candidate sequences containing patatin domain (PF01734). Similar method was used to extract the pPLA sequences of other nine species, that were used to construct evolutionary tree.

The physical and chemical properties of GhpPLA proteins, such as amino acid length, molecular weight, and isoelectric point, were analyzed using the ExPasy website (https://web.expasy.org/compute_pi/) [56] and CottonFGD. The subcellular location of candidate *GhpPLA* genes was predicted using the CELLO v2.5 server [57].

### Evolutionary relationship analysis among different species

ClustalW was used for sequence alignment in MEGA7.0 software [58], and a phylogenetic tree was constructed after sequence alignment, using the Maximum Likelihood (ML) method with 1000 bootstrap replications. To find out the genetic relationship between *G. hirsutum* and other species, two phylogenetic evolution trees were constructed. The first tree included four cotton species, *Arabidopsis*, and *Z. mays*, while the second tree encompassed *G. hirsutum*, *A. thaliana, Z. mays*, and seven additional species (*A. trichopoda, G. max, O. sativa, S. bicolor, S. moellendorffii, T. cacao, V. vinifera*).

### Chromosome mapping and collinearity analysis of *pPLAs*

Chromosome mapping of *pPLAs* in four cotton species was performed by TBtools software using gene annotation information (Gff3 format) and genome assembly sequences depending on (Fasta format) downloaded from CottonFGD data. The protein sequences of the four cotton species were used as query sequences to perform a self-blast using blastp in TBtools. The results of the blastp analysis were used to create a collinear atlas using Advanced Circos in TBtools. Blast analysis was performed between *G. hirsutum* and three different species (*A. thaliana*, *Z. mays* and *V. vinifera*) using One Step MCScanX and constructed figures by Text Merge for MCScanX.

### Analysis of the conserved protein motifs and gene structure

The MEME website (https://meme-suite.org/meme/tools/meme) [59] was utilized to detect conserved motifs in *pPLAs* gene sequences of four cotton species, with a p-value lower than 1^e − 5^, and the results were saved as a MAST file. The Batch Web CD-Search Tool in NCBI [60] was used to predict conserved domains in gene sequences, with an e-value lower than 0.01, and the hit data file was saved. TBtools was then used to combine the conserved motifs, conserved domains, phylogenetic tree, and intron/exon structure, by utilizing the nwk file, MAST file, hit data file, and gff genome files.

### Homology modeling of the 3D structure of GhpPLA proteins

The 3D crystal structure of GhpPLAs is still unknown, so the computer-based prediction was carried out through a homology modeling approach using the SWISS-MODEL website (https://swissmodel.expasy.org/) [61]. GhpPLA23, which was closely related to ZmPLA1, was selected for homology modeling. A template having the highest sequence identity and coverage to the GhpPLAs was selected from the PDB with ID: 1OXw.1B. Its sequence similarity reached 48.15%, and the model was evaluated as 0.72, showing high reliability. PDB files of the model were downloaded from SWISS-MODEL and used as input files into PrankWeb (https://prankweb.cz/) [62] to predict the active sites of protein 3D structure.

### Promoter *Cis*-element analysis and digital expression analysis of *GhpPLAs*

TBtools software was used to analyze 2000 bp DNA sequences in the upstream region of *pPLA* genes in upland cotton. Then the *cis*-regulatory elements of the promoter region from *GhpPLAs* were predicted through the PlantCARE (http://bioinformatics.psb.ugent.be/webtools/plantcare/html/) [63]. After that, *cis*-regulation elements were screened and classified, including phytohormone, plants growth and abiotic stress. To investigate the expression patterns of *GhpPLAs* across diverse tissues, RNA-Seq data (PRJNA490626) was acquired from the Cotton Omics Database (http://cotton.zju.edu.cn/). The quantification of gene expression was conducted using the fragments per kilobase of exon per million mapped (FPKM) approach. Subsequently, TBtools software was utilized to create a heat map, which was supplemented with a phylogenetic tree and *cis*-elements to provide additional information on the genes.

### qRT-PCR analysis of *pPLAs*

Six *GhpPLAs* were selected for qRT-PCR analysis, including *GhpPLA7*, *GhpPLA19*, *GhpPLA23*, *GhpPLA28*, *GhpPLA41*, and *GhpPLA44*. Different tissues were selected for qRT-PCR analysis, including sepal, bracts, petal, and anther (flowers bud size < 3 mm, 4–5 mm, 5–8 mm and > 8 mm [64, 65]). Pollen, ovule and fiber were collected from the experimental field of *G. hirsutum* cultivar Zhongmiansuo 100. Roots, stems and leaves were collected at the seedling stage of *G. hirsutum* cultivar Zhongmiansuo 100. Total RNA was extracted from these tissues using RN38-EASYspin-Plus Plant RNA Kit (AidlabCo., LTD, Beijing, China). The extracted RNA was reversely transcribed with PrimeScript™ RT reagent Kit (Takara Biomedical Technology Co., LTD, Beijing, China). Specific primers were designed for the six selected genes using qPrimerDB-qPCR primer Database websites (Additional file 6: Table S6) [66]. RT-qPCR analysis was performed using Bio-Rad 7500 fast fluorescence quantitative PCR platform with SYBR^®^ Premix Ex Taq™ (Takara Biomedical Technology Co., LTD, Beijing, China). The experiments were independently repeated three times, and the 2^–ΔΔCt^ method was used to calculate the relative expression levels of *GhpPLAs*.

### CLCrV vector construction of *GhpPLA23* and *GhpPLA44*

The specific sequence of 247 bp was selected from the CDS of the two candidate genes for the primer design of the two gene fragments. Two insertion fragments CLCrV: GhpPLA23 and CLCrV: GhpPLA44 were amplified by PCR using upland cotton pollen as a template. The vector was digested using ascI and speI as restriction sites and the restricted fragments were ligated with the digested CLCrV vector. The connected plasmids were transferred into GV3101 through transformation, the OD value of shaken strains was adjusted to 1.0 by suspension, and the cotton with two cotyledons were transformed after two hours. Transformed plants were incubated darkly in a greenhouse at 23 °C for 24 h and the yellowing phenotype was successfully observed after two weeks, indicating that VIGS was successful. After five or six months of plant growth, the flowers were picked, RNA was extracted from pollen, and qRT-PCR experiment was performed on the Bio-Rad 7500 fast fluorescence quantitative PCR platform using SYBR® Premix Ex Taq™ (Takara Biomedical Technology Co., LTD, Beijing, China). The experiments were independently repeated three times, and the 2^–ΔΔCt^ method was used to calculate the relative expression levels of *GhpPLA23* and *GhpPLA44.*

### Determination of physiological parameters of VIGS plants and observation of pollen activity

The freshly picked pollens were fixed with Carnot fixative, and then washed three times with 95% alcohol and 75% alcohol, and then washed three times with ddH_2_O. The washed pollens were dripped into the slide and stained with Alexander’s staining solution. After 4 h, the pollen viability was observed by microscope, and pollen observation of each plant was repeated three times. The pollens of the control and silenced plants were collected, cold-shocked in liquid nitrogen and stored at -80℃ for later use. Catalase (CAT), superoxide dismutase (SOD), peroxidase (POD), malonyl dialdehyde (MDA), and H_2_O_2_ activities were determined using the kits (Solarbio, Beijing, China).

### Interaction network of the pPLA proteins

STRING website (https://cn.string-db.org/) [67] was used to construct protein interaction networks for the whole family and *pPLAs* of *A.thaliana* respectively with a confidence parameter set at 0.15 threshold.

### Electronic supplementary material

Below is the link to the electronic supplementary material.

Supplementary Material 1

Supplementary Material 2

## Data Availability

The source data underlying the graphs in the main figures are available in Supplementary information. Sequence data from this work can be found in Cotton FGD (https://cottonfgd.org/) and Phytozome (https://phytozome-next.jgi.doe.gov/).

## Abbreviations

CAT: Catalase
SOD: Superoxide dismutase
POD: Peroxidase
MDA: Malonyl dialdehyde
PLAs: Phospholipases as
pPLA: Patatin-related *PLA*
JA: Jasmonic acid
Zm: *Zea mays*
Os: *Oryza sativa*
Sb: *Sorghum bicolor*
Vv: *Vitis vinifera*
Tc: *Theobroma cacao*
Gm: *Glycine max*
Atr: *Amborella trichopoda*
Sm: *Selaginella moellendorffii*
Ga: *G. arboreum*
Gr: *G. raimondii*
Gh: *G. hirsutum*
Gb: *G. barbadense*
At: *Arabidopsis thaliana*
qRT-PCR: quantitative real-time PCR
WGD: Whole genome duplication
