# Supplementary material for: Comprehensive identification and functional characterization of GhpPLA gene family in reproductive organ development
Source: BMC Plant Biol. 2023 Nov 29;23:599. doi: 10.1186/s12870-023-04590-4 (PMC10685517; doi:10.1186/s12870-023-04590-4)
Supplement: Supplementary file 2 — Supplementary Material 2 [file 12870_2023_4590_MOESM2_ESM.docx]

**Supplementary information**


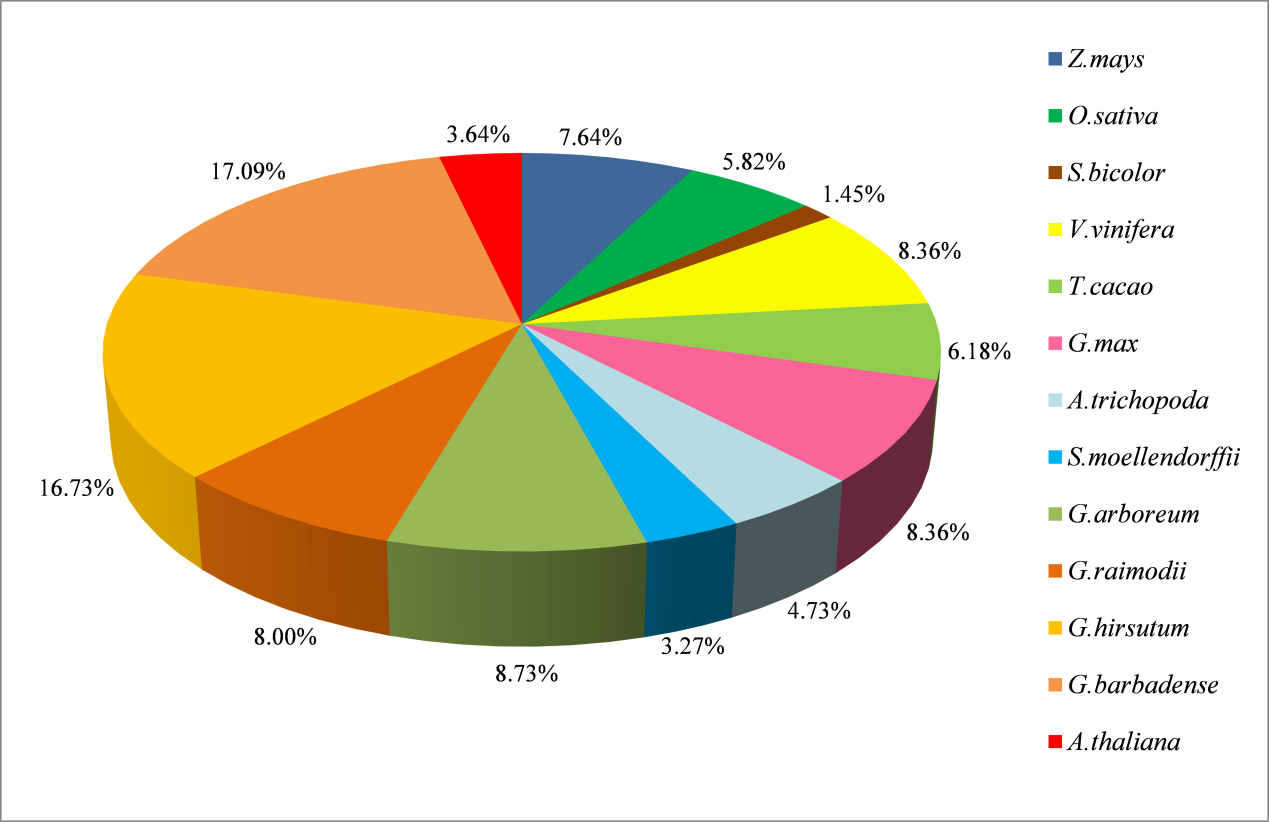


**Additional file 8: Fig. S1** Distribution of *pPLA* genes among thirteen plant species.

**
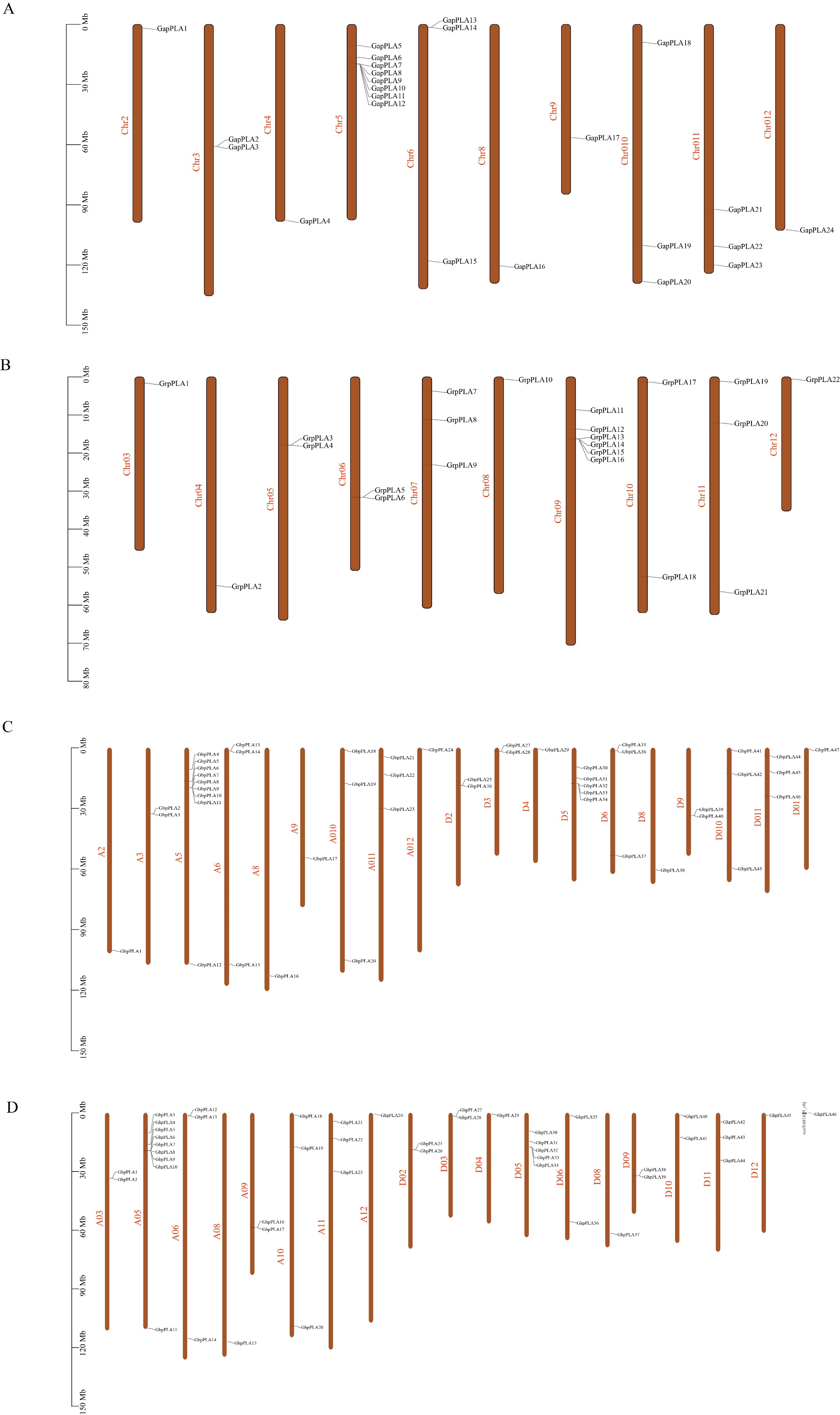
**

**Additional file 9: Fig. S2** Gene mapping of four cotton species. (A) Gene mapping of *GapPLAs*. (B) Gene mapping of *GrpPLAs*. (C) Gene mapping of *GbpPLAs*. (D) Gene mapping of *GhpPLAs*. The scale bar on the left side represents the position of the chromosome length.
